# Fellow-Workers and the Roll of Honour

**Published:** 1914-09-26

**Authors:** 


### ROUND THE WORLD.

[The Editor Invites Early Information and Contributions Marked "Fellow-Workers."]

### Fellow-Workers and the Roll of Honour.

Several well-known Cambridge men are associated with the 1st Eastern General Hospital, which has been mobilised in Neville's Court, Trinity College, Cambridge, where a number of wounded soldiers have already arrived. Sir Thomas Clifford Allbutt, M.D., K.C.B., the Regius professor of medicine at Cambridge University, and one of the most prominent figures in the medical profession in this country to-day, holds the rank of lieutenant-colonel at this hospital, where he has for colleagues of equal rank Dr. J. B. Bradbury, Dr. F. Deighton, and Mr. G. E. Wherry.

Dr. John Buckley Bradbury is Downing professor of medicine at Cambridge, and senior physician to Addenbrooke's Hospital. Studying medicine at Cambridge and King's College Hospital, he qualified M.B. in 1867, taking his Doctorate three years later. Subsequently he was elected a Fellow of the Royal College of Physicians of London, in connection with which corporation he has held the important posts of Bradshaw Lecturer, Croonian Lecturer, and member of the Council. Dr. Bradbury has written a good deal on pharmacological subjects, in addition to various articles on general medicine.

Dr. Frederick Deighton, M.B. Cantab., M.R.C.S. Eng., pursued his clinical studies at St. George's Hospital, and after qualification returned to his University town, where he became surgeon, with special reference to the gynæcological and throat and ear departments. At St. George's he held the appointments of ophthalmic and orthopædic assistant, and assistant surgical registrar. In earlier years he lectured to the probationers at Addenbrooke's Hospital.

Mr. George Wherry, M.C. Cantab., F.R.C.S. Eng., is also surgeon to Addenbrooke's Hospital, and lectures on surgery at Cambridge University. An old student of St. Thomas's Hospital, at one time he held the appointment of assistant demonstrator of anatomy there. He qualified M.R.C.S. Eng. in 1873.

Mr. H. A. Ballance, Dr. F. W. Burton-Fanning, Mr. A. Cooke, Dr. L. Humphry, Dr. P. S. Hichens, Dr. E. L. Jones, Dr. R. A. Milligan, and Dr. J. A. Wright are the majors at the 1st Eastern General Hospital.

Dr. Frederick William Burton-Fanning is an M.D. Cantab. and F.R.C.P. Lond. Studying medicine at University College Hospital, where he won the gold medal in medicine in 1885, he subsequently gave special attention to the treatment of chest diseases and settled in Norwich. Subsequently he became physician to the Norfolk and Norwich Hospital, and consulting physician to the Kelling Open-air Sanatorium, the Children's Open-air Sanatorium at Holt, and to the Cottage Hospitals at Cromer, Wells, Watton, and Swaffham. In earlier years he was associated with medical work at Cambridge, being for a while house-physician at Addenbrooke's Hospital. He is the author of valuable writings on the open-air and sanatorium treatment of consumption, in connection with which he is an acknowledged authority.

Mr. Hamilton Ashley Ballance is also a distinguished Norwich practitioner, holding the post of surgeon to the Norfolk and Norwich Hospital and consulting surgeon to several cottage hospitals in the neighbourhood. A student of University College Hospital, Mr. Ballance won many academic distinctions, including first-class honours in medicine and obstetric medicine, with the gold medal in surgery at the M.B., B.S. Lond. examination in 1892. His qualifications include those of M.S. Lond. and F.R.C.S. Eng. Mr. Ballance has seen military service before, having been surgeon to the Imperial Yeomanry Hospital during the South African War.

Mr. Arthur Cooke is an Oxford University and London Hospital man who now holds the post of assistant surgeon to Addenbrooke's Hospital. Qualifying L.R.C.P. Lond. in 1895, he graduated in medicine and surgery at Oxford in the same year, and three years later became an F.R.C.S. Eng. His earlier appointments include those of chief clinical assistant to the Royal London Ophthalmic Hospital, Moorfields, house-surgeon to the London Hospital, and demonstrator in anatomy at Oxford.

Dr. Laurence Humphry, M.D. Cantab., F.R.C.P. Lond., is one of the physicians to Addenbrooke's Hospital at Cambridge, where he was formerly a student. Pursuing his clinical studies at "Bart.'s," Dr. Humphry qualified M.R.C.S. Eng. in 1879, and subsequently became resident medical officer to the City of London Hospital for Diseases of the Chest. Apart from his work at Addenbrooke's, he is examiner in medicine at the R.A.M.C. examinations.

Dr. Peverell Smythe Hichens is a prominent member of the staff of the Northampton General Hospital, where he holds the post of honorary physician; he is also consulting physician to the Northamptonshire Sanatorium, examining physician to Queen Alexandra's Hospital at Davos, and medical referee to the Royal National Hospital for Consumption. Qualifying M.B., B.Ch. from St. Thomas's in 1895, Dr. Hichens subsequently took his doctor's degree, and was elected an F.R.C.P. Lond. in 1911. From early years he has been interested in tuberculosis, and soon after qualification held the post of assistant resident medical officer at the Brompton Consumption Hospital.

Dr. Ernest Lloyd Jones joined Cambridge University with a Clothworkers' Scholarship in 1882, and four years later entered St. Bartholomew's with the Senior Science Scholarship. Qualifying M.B. Cantab. in 1888, he held resident appointments at St. Bartholomew's Hospital, the Royal Hospital for Diseases of the Chest, and the Western General Dispensary. Taking his M.D. Cantab. in 1891, he eventually settled in Cambridge, where he has had a distinguished career, holding now the appointments of assistant physician to Addenbrooke's Hospital and demonstrator of medicine in the University.

Dr. W. W. Rorke, the hon. pathologist and radiographer to the Walthamstow Hospital, is, we are informed, working in one of the base hospitals in England.

